# Interplay of coupling and common noise at the transition to synchrony in oscillator populations

**DOI:** 10.1038/srep38518

**Published:** 2016-12-06

**Authors:** Anastasiya V. Pimenova, Denis S. Goldobin, Michael Rosenblum, Arkady Pikovsky

**Affiliations:** 1 Institute of Continuous Media Mechanics, UB RAS, Perm 614013, Russia; 2 Department of Theoretical Physics, Perm State University, Perm 614990, Russia; 3 Institute for Physics and Astronomy, University of Potsdam, Karl-Liebknecht-Str. 24/25, 14476 Potsdam-Golm, Germany; 4 Department of Control Theory, Nizhny Novgorod State University, Gagarin Av. 23, 606950, Nizhny Novgorod, Russia

## Abstract

There are two ways to synchronize oscillators: by coupling and by common forcing, which can be pure noise. By virtue of the Ott-Antonsen ansatz for sine-coupled phase oscillators, we obtain analytically tractable equations for the case where both coupling and common noise are present. While noise always tends to synchronize the phase oscillators, the repulsive coupling can act against synchrony, and we focus on this nontrivial situation. For identical oscillators, the fully synchronous state remains stable for small repulsive coupling; moreover it is an absorbing state which always wins over the asynchronous regime. For oscillators with a distribution of natural frequencies, we report on a counter-intuitive effect of dispersion (instead of usual convergence) of the oscillators frequencies at synchrony; the latter effect disappears if noise vanishes.

Synchronization effects in ensembles of coupled oscillators are relevant for various physical systems, such as coupled lasers, spin-torque oscillators, and Josephson junctions^1^,^2^,^3^, as well as for diverse natural phenomena in life sciences^4^,^5^, and even for many social systems^6^,^7^. Synchronization caused by an attractive mean-field coupling, studied in pioneering works by Winfree and Kuramoto^8^,^9^, allows a two-fold characterization. On one hand, the synchronization transition can be described via the appearance of a macroscopic mean field, amplitude of which often serves as the order parameter of the transition. On the other hand, synchronization can be characterized via an adjustment of the frequencies of the oscillators in the ensemble (e. g., N. Wiener described synchronization^10^ as a “phenomenon of the pulling together of frequencies”). There is also a nontrivial way to synchronize oscillators without coupling by acting on them with a common external noise^11^,^12^,^13^,^14^,^15^,^16^. Weak noise can be treated in the phase approximation, and here only synchronization is possible. For strong noise, one has to go beyond the phase approximation, and here desynchronization can occur if the oscillators are strongly nonisochronous^13^,^17^. Remarkably, common noise synchronizes oscillators in the first meaning only. So, an ensemble of identical uncoupled oscillators under common noise forms a perfect cluster where all the states coincide and the value of the order parameter is the maximal possible. The phases of slightly different oscillators form an imperfect cluster; their frequencies are however not adjusted: their difference is preserved under common noise.

In this paper we study properties of synchronization and of the behavior of the frequencies if both coupling and common noise are present. We consider phase oscillators, what corresponds to the situation of small noise, so that noise always tends to synchronize the ensemble. This setup corresponds to experiments with metronomes on a common support^18^, if the latter is subject to a random force, and to experiments with coupled electronic oscillators^19^,^20^ with an additional driving noisy current. Our theory generalizes previous studies of noise-driven ensembles with^21^,^22^ and without coupling^23^ (dynamics of two oscillators with coupling and common and intrinsic noises has been studied in ref. 24). Similar to refs 22, 23 we use the Ott-Antonsen ansatz allowing one to get a closed stochastic equation for the order parameter of the ensemble. This ansatz is valid in the thermodynamic limit for coupled oscillators with a Lorenzian distribution of natural frequencies, and it can be generalized to include a common noisy driving. After averaging of the resulting equations over the fast basic frequency of oscillations, we get a tractable Langevin-type dynamics of the order parameter. We discuss in detail a nontrivial competition between the synchronizing action of noise and the desynchronizing action of the repulsive coupling. Here our study goes beyond that of ref. 22, which has been mainly focused on the effects of noise on the properties of the ensemble close to the transition to synchrony for attractive coupling. For nonidentical oscillators, where complete synchrony is impossible, we derive stationary distribution of the order parameter and describe a rather counter-intuitive dispersion of the frequencies at synchronization in presence of the repulsive coupling. This appears to be a surprising state where “phase locking” is accompanied by “frequency anti-entrainment”: although the oscillator phases are most of the time close to each other, their frequencies become more diverse compared to natural ones.

## Results

### Basic equations

We consider an ensemble of phase oscillators subject to a common Gaussian white noise with intensity *σ*^2^ and to a Kuramoto-type coupling with strength *μ* (the coupling is attractive for *μ* > 0 and repulsive otherwise). We consider the ensemble in the thermodynamic limit, suitable for the application of the Ott-Antonsen theory^25^:


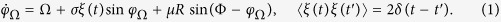


Here the mean field is defined as


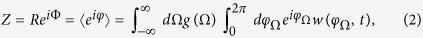


where *g*(Ω) is the distribution of the natural frequencies. According to the Ott-Antonsen ansatz^25^, the distribution function of the phases at given Ω can be represented as 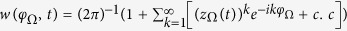 and the mean field *z*_Ω_(*t*) of a subpopulation with frequency Ω obeys the equation


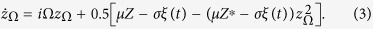


For a Lorentzian distribution of frequencies *g*(Ω) = *γ*[*π*(*γ*^2^ + (Ω − Ω_0_)^2^)]^−1^, the integral in (2) can be calculated by virtue of the residual theorem, under assumption of analyticity of *z*_Ω_ in the upper half-plane, 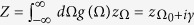. As a result one obtains a closed equation for the mean field *Z* for coupled non-identical oscillators under common noise:


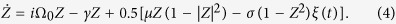


It contains four parameters: the basic frequency Ω_0_ (which, in contradistinction to the usual Kuramoto model, cannot be simply shifted to zero, because the noise term breaks the frequency-shift invariance), the noise intensity *σ*^2^, the coupling constant *μ*, and the width of the distribution of natural frequencies *γ*.

Generally, the common noise can not only synchronize identical oscillators, but also desynchronize them provided nonisochronicity inheres in the systems and the noise is strong enough to evoke significant perturbations of the amplitude degrees of freedom^11^,^12^,^13^. However, in the systems with purely phase dynamics, like equation (1) we consider in this paper, the noise can make only the synchronizing effect^14^,^15^; the effect of a weak noise is also generally synchronizing^16^.

For an analytical treatment below, it is convenient to use the real-valued variables (*J*, Φ), where *J* = *R*^2^/(1 − *R*^2^) is another order parameter characterizing the level of synchrony (closeness of the phases of oscillators in the ensemble): for *J* = 0 the mean field amplitude 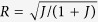 vanishes, while the full synchrony with *J* = ∞ corresponds to *R* = 1. Equations for these variables read


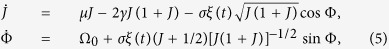


and are complemented with the equation for the phase, relative to that of the mean field, *θ*_*ω*_ = *ϕ*_Ω_ − Φ:


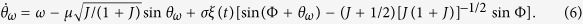


Here *ω* = Ω − Ω_0_ is the deviation of the natural frequency from the ensemble mean. For the sake of simplicity of notations we omit index *ω* below.

### Averaged equations

As the first step, we employ the natural condition that the basic frequency of oscillations Ω_0_ is much larger than the parameters *μ*, *γ*, *σ*^2^ (which all have dimension of inverse time). This suggests averaging over the fast rotating phase Φ. One writes the Fokker-Planck equation corresponding to the Langevin equations (5, 6), and by virtue of the multiple scales expansion (see Section 1 of Supplementary material for a detailed derivation) obtains in the leading order in the small parameters *μ*, *γ*, *σ*^2^ the following equation for the probability density *w*(*J*, *θ*, *t*):


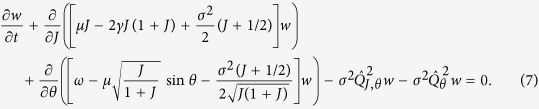


Here we defined the operators


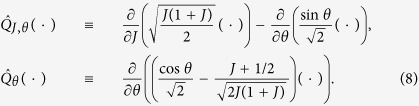


The Fokker-Planck equations (7, 8) is equivalent to the following system of stochastic Langevin equations which can be interpreted as Eqs (5, 6) averaged over the fast oscillations with frequency Ω_0_:


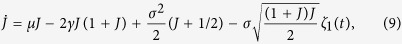



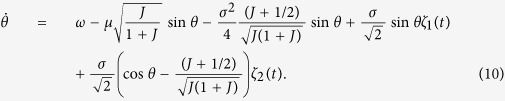


The original noise *ξ*(*t*) generates two effective independent noise terms *ζ*_1_(*t*) and *ζ*_2_(*t*), which are Gaussian and delta-correlated, 〈*ζ*_*n*_(*t*)*ζ*_*l*_(*t* + *t*′)〉 = 2*δ*_*n*,*l*_*δ*(*t*′), because the signals *ξ*(*t*) cos Ω_0_*t* and *ξ*(*t*) sin Ω_0_*t* are uncorrelated on time scales that are large compared to 2*π*/Ω_0_. The derived equations contain four parameters *μ*, *γ*,* σ*^2^, *ω*, and the properties of the stationary solutions depend on *μ*/*σ*^2^, *γ*/*σ*^2^, and *ω*/*σ*^2^ only.

Our first goal is to characterize the statistics of the order parameter *J*. One can see that, as it should be for any global coupling setup, the system (9,10) is a skew one, where the dynamics of the order parameter affects that of the phases, but not vice versa. Thus one obtains a closed Fokker-Planck equation (the corresponding Langevin equation is (9)) for the distribution of the order parameter


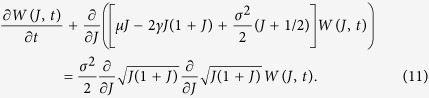


### Identical oscillations

We start by considering the case of identical oscillators *γ* = 0. Here, the analysis of states close to full synchrony *J* → ∞ is simple, as ln *J* performs a biased random walk:


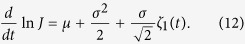


The quantity 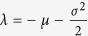 is nothing else as the Lyapunov exponent determining stability of the full synchrony, the latter is stable if *λ* < 0, i.e. if *μ* > −*σ*^2^/2. Thus, the small enough repulsive coupling between the oscillators does not break stability of the full synchrony. Another important state is that of full asynchrony, *J* = 0. One can see however from Eq. (9) that this state is not invariant in presence of noise.

In fact, here we meet a nontrivial situation where the states of full asynchrony (*J* = 0) and of full synchrony (*J* = ∞) are differently driven by noise. For the asynchronous state the driving is additive, therefore this state is not invariant and the order parameter experience fluctuations close to *J* = 0, even if this state is stable (i.e. for repulsive coupling *μ* < 0). In contradistinction, the noise is acting on the fully synchronous state in a multiplicative way, so that if this state is stable, noise does not kick the system out of it. Thus, the stable (*λ* < 0) fully synchronous state is an absorbing one. This means that also for a slightly repulsive coupling −*σ*^2^/2 < *μ* < 0, the asynchronous state *J* ≈ 0, although stable without noise, does not survive the competition with the fully synchronous state *J* = ∞ which is the global attractor.

In this “bistable” situation the nontrivial statistical characteristic is the mean first passage time 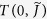 for the stochastic process (9,11), from asynchrony *J*(0) = 0 to synchrony 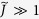 (here a cutoff is needed, because the approach to the full synchrony *J* = ∞ is exponential, formally the time to achieve it is infinite). The expression for *T* can be found via the standard first-passage time theory for one-dimensional stochastic processes^26^ (see Section 2 of Supplementary material for details):


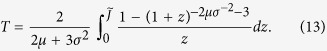


Depending on the value of *μ*/*σ*^2^, this time changes from a logarithmically large one 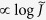 for 2*μσ*^−2^ + 3 > 0, to a time diverging as a power law of 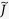 for 2*μσ*^−2^ + 3 < 0.

### Nonidentical oscillators

For nonidentical oscillators, *γ* > 0, the fully synchronous state does not exist. In this situation the order parameter *J* fluctuates with the stationary distribution, which can be straightforwardly found from (11):


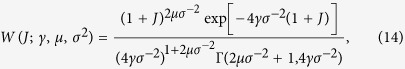


where Γ(*m*, *x*) is the upper incomplete Gamma function. The average value of the order parameter is


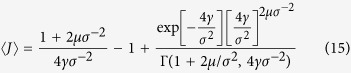


**Figure 1:**
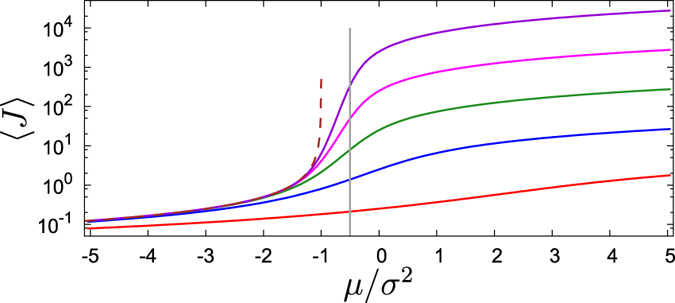
Values of 〈*J*〉 according to (15) for different *γ*/*σ*^2^ as functions of *μ*/*σ*^2^. From top to bottom: *γ*/*σ*^2^ = 10^−4^, 10^−3^, 10^−2^, 10^−1^, 1. Brown dashed line corresponds to the system of identical oscillators *γ* = 0. Vertical grey line shows the border of stability of the fully synchronous state for *γ* = 0.

These expressions are valid for any *γ* > 0, however the limit *γ* → 0 is singular: a normalizable distribution for *J* at *γ* = 0


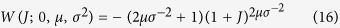


exists only if the synchronous state is unstable, i.e. *μ* < −*σ*^2^/2, and the average 〈*J*〉 = −(2*μσ*^−2^ + 2)^−1^ is finite only if *μ* < −*σ*^−2^. We present the dependencies of 〈*J*〉 on the parameters of the problem in Fig. 1.

For nonidentical oscillators we face a new problem of the behavior of the frequencies. The skew Langevin Eqs (9,10) appear to be analytically solvable only if we make another approximation: We neglect fluctuations of the order parameter (i.e. we assume *J* ≈ *const*, for large *J* this agrees with numerics) in the equations for the phases. In this approximation we obtain from Eq. (10) a closed Langevin equation for the phase dynamics:


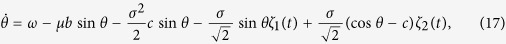


where we denote 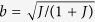, 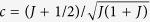. The stationary solution *w*(*θ*) of the corresponding Fokker-Planck equation with a constant flux 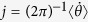 obeys


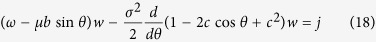


(see Section 3 of Supplementary material for details). Solution of this equation reads


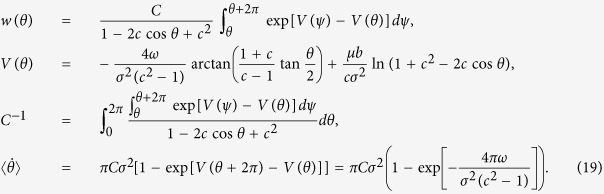


This rather lengthy exact solution can be simplified, for small *ω*, to include the first-order terms ~*ω* only. Here the expression for *j* reduces to 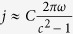, and in the normalization factor *C* we can set *ω* = 0:


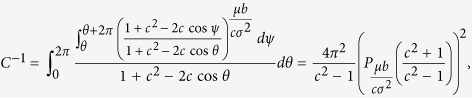


where *P*_*λ*_(*x*) is the Legendre function. A rather simple expression appears for small *μ*, where an expansion of the Legendre function can be used. The final approximate formula for the observed frequencies of oscillators 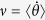 reads


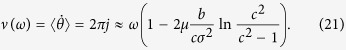


**Figure 2:**
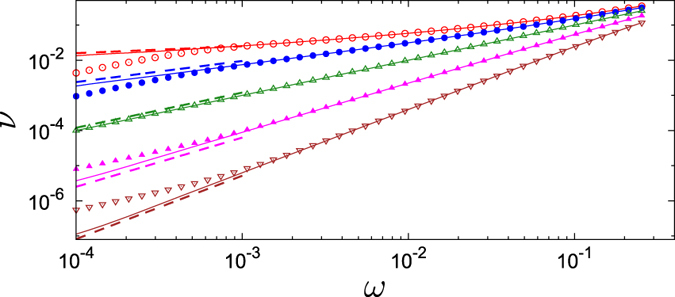
Observed frequencies *ν* vs natural frequencies *ω*, obtained from (19). We use here the continuous fraction expansion of the Fourier representation of *w*(*θ*), following^27^ (see Section 3 of Supplementary material for details). Solid lines: solutions for *J* = ∞, markers: solutions for 〈*J*〉 = 10. From top to bottom: *μ*/*σ*^2^ = −0.4, −0.2, 0, 0.2, 0.4. Dashed lines have slopes 1 + 2*μ*/*σ*^2^.

Noteworthy, for uncoupled oscillators (*μ* = 0) one obtains *ν* = *ω*. This means that common noise does not influence the average frequencies. In the presence of coupling, the observed frequencies *ν* are pulled together if the coupling is attractive, *μ* > 0, and are pushed apart if the coupling is repulsive, *μ* < 0. The effect depends on the level of synchrony, characterized by the value of the order parameter *J*. In fact, the limit *J* → ∞ is singular as here *c* → 1; as we show in Fig. 2, in this limit the dependence *ν*(*ω*) is not linear, but a power law one. Unfortunately, it is difficult to confirm these curves by direct simulations of the original system, because here we assume a definite value of the order parameter, which in a practical situation depends in a non-trivial way on the parameters of the problem – noise, coupling, and spread of natural frequencies.

Formula (21) describes, in an approximate way, the main nontrivial effect that appears due to combined action of common noise and mean-field coupling on the ensemble of nonidentical oscillators. We first remind what happens to the frequencies in the absence of the common noise, i.e. for the standard Kuramoto model. In this case there is a critical value of the coupling constant, beyond which the order parameter is non-zero. In this synchronized state the frequencies are pulled together; moreover there appears a cluster of oscillators that have equal frequencies, the size of this cluster grows with the coupling constant. Below the critical coupling strength, the order parameter vanishes, so that there is no effect on the frequencies of the oscillators, and they remain the natural ones.

Common noise additionally influences the order parameter, which is non-vanishing and even large also when the mean-field coupling is repulsive (cf. Fig. 1). This leads to a surprising state of synchronization with dispersion of the frequencies: synchrony (in the sense of a large value of the order parameter) is in this case maintained by the common noise, while the repulsive coupling is responsible for the scattering of frequencies.

As this effect is notable, we characterize it below numerically on different levels. First, in Fig. 2 we show the solutions (19) for *J* = ∞ (perfect synchronization) and for a finite *J*. One can see that in the fully synchronous case *J* = ∞ the repulsion of frequencies is not linear as in Eq. (21), but follows a power law *ν* ∝ *ω*^*α*^, with an exponent that with high accuracy can be fitted as *α* = 1 + 2*μ*/*σ*^2^.

**Figure 3:**
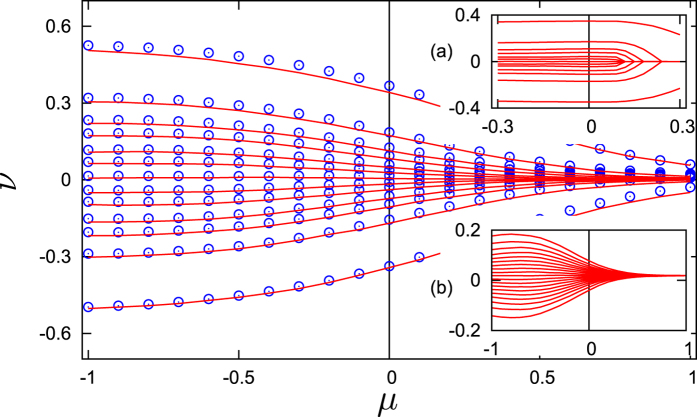
Observed frequencies *ν* vs coupling strength *μ*, obtained from (1,4). Parameters of simulations: Ω_0_ = 100, *σ* = 1, *γ* = 0.05. Markers: direct simulations of the population of 21 phase oscillators (1) (for better visibility, not all frequencies are depicted), solid lines: simulations of the Ott-Antonsen equations (4), valid in the thermodynamic limit, for the same individual frequencies. The inset (**a**) shows the case without noise *σ* = 0. The inset (**b**) shows the case of a Gaussian distribution of frequencies.

Next, we illustrate in Fig. 3 the effect of dispersion of the frequencies with the direct simulation of Langevin equations (1,4) describing the ensemble of coupled oscillators. One clearly sees dispersion of the frequencies for the repulsive coupling and their concentration for the attractive coupling, both for the cases of Ott-Antonsen equations (4) valid in the thermodynamic limit, and for a finite population governed by (1). This should be contrasted to the ensemble without noise (inset (a) in Fig. 3) where for repulsive coupling there is no repulsion of frequencies. Additionally, we confirm in inset (b) in Fig. 3 that the effect is valid not only for the Lorentzian distribution, but also for the Gaussian one.

## Discussion

To summarize, in this paper we have developed a theory for an ensemble of coupled oscillators driven by common noise. In the thermodynamic limit, by adopting the Ott-Antonsen ansatz and by averaging over the high basic frequency, we obtain analytically tractable equations for the order parameter and find the distribution of the order parameter in a closed form. As the common noise always fosters synchrony of oscillators, nontrivial features appear if the mean-field coupling acts in the opposite direction, i.e. is repulsive. For identical oscillators this competition results in the existence of the critical coupling strength *μ*_*c*_ = −*σ*^2^/2. For *μ* > *μ*_*c*_ the fully synchronous state where all the oscillators form a perfect cluster is stable, while for *μ* < *μ*_*c*_ it is not. Because, for vanishing noise, the splay state with a uniform distribution of phases is stable for all negative values of *μ*, one could expect bistability for *μ*_*c*_ < *μ* < 0. However, bistability does not happen, because the noise acts differently at the two states of interest: it is additive for the splay state with vanishing order parameter, and is multiplicative for the fully synchronous state. The latter is thus an absorbing state and the system never leaves it when the full synchrony is achieved. Therefore for *μ*_*c*_ < *μ* only the synchronous state is eventually observed, and the only nontrivial question is how fast it is reached—the answer to this question is given by Eq. (13).

Another quite counter-intuitive effect of the competition between the common noise and the coupling can be observed for non-identical oscillators. The order parameter is always non-vanishing in presence of common noise, and this leads to dispersion of the frequencies—their distribution is wider than in the coupling-free case. Here one should take into account that the common noise does not directly adjust the frequencies, although it pulls the phases together into a stochastic bunch. In presence of an additional repulsive coupling, the phases in the bunch repel each other (although synchrony is preserved) and as the result their frequencies diverge. This implies that in such a state, phase locking (characterized by large values of order parameter) is accompanied by frequency anti-entrainment (characterized by average frequencies). For example, many pendulum clocks (or metronomes) on a randomly forced platform will look like synchronous in snapshots of the pendula positions, while their average periods will differ stronger than without coupling and/or noise.

## Methods

Numerical methods have been used to prepare Figs 2 and 3. Solution of the Fokker-Planck equation (18) was accomplished by virtue of an expansion of the solution in the Fourier series, and by solution of the resulting linear system of equation by virtue of the continuous fraction expansion method described in ref. 27. Results presented in Fig. 3 have been obtained by virtue of numerical simulation of stochastic differential equations using the stochastic Runge-Kutta method described in ref. 28; further details on the direct simulation can be found in Section 4 of Supplementary material.

## Additional Information

**How to cite this article**: Pimenova, A. V. *et al*. Interplay of coupling and common noise at the transition to synchrony in oscillator populations. *Sci. Rep.* **6**, 38518; doi: 10.1038/srep38518 (2016).

**Publisher's note:** Springer Nature remains neutral with regard to jurisdictional claims in published maps and institutional affiliations.

## Supplementary Material

Supplementary Information
