# Author Correction: Interplay of coupling and common noise at the transition to synchrony in oscillator populations

**DOI:** 10.1038/s41598-023-35268-4

**Published:** 2023-05-23

**Authors:** Anastasiya V. Pimenova, Denis S. Goldobin, Michael Rosenblum, Arkady Pikovsky

**Affiliations:** 1 Institute of Continuous Media Mechanics, UB RAS, Perm, 614013 Russia; 2 Department of Theoretical Physics, Perm State University, Perm, 614990 Russia; 3 Institute for Physics and Astronomy, University of Potsdam, Karl-Liebknecht-Str. 24/25, 14476 Potsdam-Golm, Germany; 4 Department of Control Theory, Nizhny Novgorod State University, Gagarin Av. 23, Nizhny Novgorod, 606950 Russia

Correction to: *Scientific Reports* 10.1038/srep38518, published online 06 December 2016

This Article contains an error in Equation 13 and the sentence following this equation. The mean first passage time was originally calculated from the backward Fokker–Planck equation (or Kolmogorov equation) corresponding to the Ito interpretation of the Langevin equation for the order parameter, while this Langevin equation was derived and treated in the Stratonovich interpretation. The corrected first passage time problem yields an asymptotic behaviour with the same critical coupling strength as the analysis of the Lyapunov exponent.

Therefore,

$$T=\frac{2}{2\mu +{3\sigma }^{2}}{\int }_{0}^{\widetilde{J}}\frac{1-{\left(1+z\right)}^{-2\mu {\sigma }^{-2}-3}}{z}dz.$$

Depending on the value of $\mu /{\sigma }^{2}$, this time changes from a logarithmically large one $\propto \mathrm{log}\widetilde{J}$ for $2\mu {\sigma }^{-2}+3>0$, to a time diverging as a power law of $\widetilde{J}$ for $2\mu {\sigma }^{-2}+3<0$.

should read:

$$T=\frac{2}{2\mu +{\sigma }^{2}}{\int }_{0}^{\widetilde{J}}\frac{1-{\left(1+z\right)}^{-2\mu {\sigma }^{-2}-1}}{z}dz.$$

Depending on the value of $\mu /{\sigma }^{2}$, this time changes from a logarithmically large one $\propto \mathrm{log}\widetilde{J}$ for $2\mu {\sigma }^{-2}+1>0$, to a time diverging as a power law of $\widetilde{J}$ for $2\mu {\sigma }^{-2}+1<0$.

As a result, the Supplementary Information file that accompanies this Article contains errors in Section II ‘TRANSITION TO THE SYNCHRONOUS STATE FOR IDENTICAL OSCILLATORS: FIRST PASSAGE TIME’ where the Equations 9, 10, 11 and the sentences following these equations respectively, are affected.

The corrected Supplementary Information file is linked to this correction notice.

## Supplementary Information

Supplementary Information.

