# Supplementary material for: Interplay of coupling and common noise at the transition to synchrony in oscillator populations
Source: Sci Rep. 2016 Dec 6;6:38518. doi: 10.1038/srep38518 (PMC5138633; doi:10.1038/srep38518)
Supplement: Supplementary Information [file srep38518-s1.pdf]

Supplementary material for the paper "Interplay of coupling and common noise at the transition to synchrony in oscillator populations"

Anastasiya V. Pimenova,[1] Denis S. Goldobin,[1,2] Michael Rosenblum,[3] and Arkady Pikovsky[3,4]
[1]Institute of Continuous Media Mechanics, UB RAS, 614013 Perm, Russia
[2]Department of Theoretical Physics, Perm State University, 614990 Perm, Russia
[3]Institute for Physics and Astronomy, University of Potsdam, 14476 Potsdam-Golm, Germany
[4]Department of Control Theory, Nizhny Novgorod State University, 606950 Nizhny Novgorod, Russia

(Dated: October 28, 2016)

I. AVERAGING OF EQUATIONS OVER FAST NATURAL OSCILLATIONS IN THE PRESENCE OF COMMON NOISE

In this section we derive the Fokker–Planck equation and the effective equations for the dynamics of the order parameter J and the phase shift $\theta_\omega = \varphi_\Omega - \Phi$ for the case of a high natural frequency and nonidentical oscillators. We start with the equation system (5)–(6) of the main paper:

$$\dot{J} = \mu J - 2\gamma J(1+J) - \sigma\xi(t)\sqrt{J(1+J)}\cos\Phi, \tag{1}$$

$$\dot{\Phi} = \Omega_0 + \sigma\xi(t)\frac{J+1/2}{\sqrt{J(1+J)}}\sin\Phi, \tag{2}$$

$$\dot{\theta}_\omega = \omega - \mu\sqrt{\frac{J}{1+J}}\sin\theta_\omega + \sigma\xi(t)\left(\sin(\Phi+\theta_\omega) - \frac{J+1/2}{\sqrt{J(1+J)}}\sin\Phi\right), \tag{3}$$

where $\omega = \Omega - \Omega_0$ and $\xi(t)$ is a normalized Gaussian delta-correlated noise: $\langle\xi(t)\rangle = 0$ and $\langle\xi(t)\xi(t+t')\rangle = 2\delta(t')$. For the sake of simplicity of notations we omit the subscript ω below.

The Fokker–Planck equation for the time-dependent probability density $\rho(J,\Phi,\theta,t)$ of the states of stochastic system (1)–(3) reads

$$\frac{\partial}{\partial t}\rho + \frac{\partial}{\partial J}\Big[\left(\mu J - 2\gamma J(1+J)\right)\rho\Big] + \frac{\partial}{\partial\Phi}\Big[\Omega_0\rho\Big] + \frac{\partial}{\partial\theta}\left[\left(\omega - \mu\sqrt{\frac{J}{1+J}}\sin\theta\right)\rho\right] - \sigma^2\hat{Q}^2\rho = 0\,, \tag{4}$$

where

$$\hat{Q}(\cdot) \equiv \frac{\partial}{\partial J}\Big(-\sqrt{J(1+J)}\cos\Phi\,(\cdot)\Big) + \frac{\partial}{\partial\Phi}\left(\frac{J+1/2}{\sqrt{J(1+J)}}\sin\Phi\,(\cdot)\right) + \frac{\partial}{\partial\theta}\left(\left(\sin(\Phi+\theta) - \frac{J+1/2}{\sqrt{J(1+J)}}\sin\Phi\right)(\cdot)\right).$$

For vanishing μ, σ, and γ, the probability density distribution $\rho(J,\Phi,\theta,t) = (2\pi)^{-1}w(J,\theta)$, where

$$\int_0^{+\infty}\mathrm{d}J\int_0^{2\pi}\mathrm{d}\theta\,w(J,\theta) = 1\,.$$

Hence, for $\mu \sim \sigma^2 \sim \gamma \ll \Omega_0$, one can assume $\mu = \sigma^2\mu_1$, $\gamma = \sigma^2\gamma_1$, $\omega = \sigma^2\omega_1$ and employ the standard method of multiple scales [A.H. Nayfeh, *Introduction to Perturbation Techniques* (Wiley-VCH, 2011)]; $t_n = \sigma^{2n}t$ and $\rho = \rho^{(0)}(J,\theta,t_1,t_2,...) + \sigma^2\rho^{(1)}(J,\Phi,\theta,t_0,t_1,t_2...) + \dots$. To the leading order, Eq. (4) yields $\rho^{(0)} = (2\pi)^{-1}w(J,\theta,t_1,t_2,...)$. In the order σ^2, Eq. (4) yields

$$\frac{\partial\rho^{(1)}}{\partial t_0} + \Omega_0\frac{\partial\rho^{(1)}}{\partial\Phi} + \frac{\partial\rho^{(0)}}{\partial t_1} + \frac{\partial}{\partial J}\Big[\big(\mu_1 J - 2\gamma_1 J(1+J)\big)\rho^{(0)}\Big] + \frac{\partial}{\partial\theta}\left[\left(\omega_1 - \mu_1\sqrt{\frac{J}{1+J}}\sin\theta\right)\rho^{(0)}\right] - \hat{Q}^2\rho^{(0)} = 0\,.$$

Integrating the latter equation over Φ from 0 to 2π, one finds

$$\frac{\partial}{\partial t_0}\int_0^{2\pi}\rho^{(1)}\mathrm{d}\Phi + \frac{\partial w(J,\theta,t_1)}{\partial t_1} + \frac{\partial}{\partial J}\Big[\big(\mu_1 J - 2\gamma_1 J(1+J)\big)w(J,\theta,t_1)\Big]$$
$$+ \frac{\partial}{\partial\theta}\left[\left(\omega_1 - \mu_1\sqrt{\frac{J}{1+J}}\sin\theta\right)w(J,\theta,t_1)\right] - \frac{1}{2\pi}\int_0^{2\pi}\hat{Q}^2 w(R,\theta,t_1)\,\mathrm{d}\Phi = 0\,.$$

To eliminate the linear growth of $\rho^{(1)}$ with t_0, which would break the hierarchy of smallness of expansion terms, one has to set the first term to zero. After laborious but straightforward calculations, one can evaluate the integral

$$\frac{1}{2\pi}\int_0^{2\pi}\mathrm{d}\varPhi\,\hat{Q}^2 w(J,t_1)=\frac{\partial}{\partial J}\left(-\frac{J+1/2}{2}w\right)+\frac{\partial}{\partial\theta}\left(\frac{\sin\theta}{2}\frac{J+1/2}{\sqrt{J(1+J)}}w\right)+\hat{Q}^2_{J,\theta}w+\hat{Q}^2_{\theta}w\,,$$

where

$$\hat{Q}_{J,\theta}(\cdot)\equiv\frac{\partial}{\partial J}\Big(-\sqrt{\frac{J(1+J)}{2}}\,(\cdot)\Big)+\frac{\partial}{\partial\theta}\left(\frac{\sin\theta}{\sqrt{2}}\,(\cdot)\right),\qquad \hat{Q}_{\theta}(\cdot)\equiv\frac{\partial}{\partial\theta}\left(\left(\frac{\cos\theta}{\sqrt{2}}-\frac{J+1/2}{\sqrt{2J(1+J)}}\right)(\cdot)\right).$$

Thus, the probability density $w(J,\theta,t)$ is governed by the equation

$$\frac{\partial w}{\partial t}+\frac{\partial}{\partial J}\Big(\Big[\mu J-2\gamma J(1+J)+\frac{\sigma^2}{2}(J+1/2)\Big]w\Big)$$
$$+\frac{\partial}{\partial\theta}\left[\left(\omega-\mu\sqrt{\frac{J}{1+J}}\sin\theta-\frac{\sigma^2}{2}\frac{J+1/2}{\sqrt{J(1+J)}}\sin\theta\right)w\right]-\sigma^2\hat{Q}^2_{J,\theta}w-\sigma^2\hat{Q}^2_{\theta}w=0\,. \tag{5}$$

Integrating of the latter equation over θ, one can finds the Fokker–Planck equation for the probability density of the order parameter $W(J,t)=\int_0^{2\pi}w(J,\theta,t)\,\mathrm{d}\theta$;

$$\frac{\partial W(J,t)}{\partial t}+\frac{\partial}{\partial J}\left(\Big[\mu J-2\gamma J(1+J)+\frac{\sigma^2}{2}(J+1/2)\Big]W(J,t)\right)=\frac{\sigma^2}{2}\frac{\partial}{\partial J}\sqrt{J(1+J)}\frac{\partial}{\partial J}\sqrt{J(1+J)}\,W(J,t)\,. \tag{6}$$

Eq. (5) can be treated as the Fokker–Planck equation for the stochastic equation system with two independent noise signals $\zeta_1(t)$ and $\zeta_2(t)$;

$$\dot{J}=\mu J-2\gamma J(1+J)+\frac{\sigma^2}{2}(J+1/2)-\sigma\sqrt{\frac{J(1+J)}{2}}\zeta_1(t)\,, \tag{7}$$

$$\dot{\theta}=\omega-\mu\sqrt{\frac{J}{1+J}}\sin\theta-\frac{\sigma^2}{2}\frac{J+1/2}{\sqrt{J(1+J)}}\sin\theta+\sigma\frac{\sin\theta}{\sqrt{2}}\zeta_1(t)+\sigma\left(\frac{\cos\theta}{\sqrt{2}}-\frac{J+1/2}{\sqrt{2J(1+J)}}\right)\zeta_2(t)\,. \tag{8}$$

The original noise $\xi(t)$ generates two independent efficient noise signals $\zeta_1(t)$ and $\zeta_2(t)$, which are Gaussian and delta-correlated, $\langle\zeta_n(t)\zeta_l(t+t')\rangle=2\delta_{n,l}\delta(t')$, as the signals $\xi(t)\cos\varOmega_0 t$ and $\xi(t)\sin\varOmega_0 t$ are uncorrelated on time scales large compared to $2\pi/\varOmega_0$.

Eqs. (7) and (8) are identical to Eqs. (9) and (10) of the main paper. The Fokker–Planck equation (6) is identical to Eq. (11) of the main paper.

II. TRANSITION TO THE SYNCHRONOUS STATE FOR IDENTICAL OSCILLATORS: FIRST PASSAGE TIME

For the ensemble of identical oscillators the perfect synchrony state becomes possible. As the system can approach the perfect synchrony state only asymptotically, the transition time is infinite. However, one can consider how the system approaches the perfect synchrony state and pose the problem of the first passage time for some large value J. For Eq. (7) [or Eq. (6)], the first passage time $T(J_0,J)$ from J_0 to J obeys [C.W. Gardiner, *Handbook of Stochastic Methods* (Springer, Berlin, 1996)]

$$B(J_0)\frac{\partial^2 T(J_0,J)}{\partial J_0^2}+A(J_0)\frac{\partial T(J_0,J)}{\partial J_0}=-1\,, \tag{9}$$

with $T(J,J)=0$, $\big(\partial T(J_0,J)/\partial J_0\big)|_{J_0=J}=0$, and

$$A(J_0)=\mu J_0+\frac{\sigma^2}{2}(J_0+1/2),\qquad B(J_0)=\frac{\sigma^2}{2}J_0(1+J_0)\,.$$

In our problem $J_0 = 0$ is the boundary of the domain of possible states of the system. The solution to Eq. (9) is

$$T(J_0, J) = \int_{J_0}^{J} \mathrm{d}J_1 \int_{J_0}^{J_1} \frac{\mathrm{d}J_2}{B(J_2)} e^{-\int\limits_{J_2}^{J_1} \frac{A(J_3)}{B(J_3)}\mathrm{d}J_3} .$$

Substituting $A(J_0)$ and $B(J_0)$, one can find

$$T(J_0, J) = \frac{2}{\sigma^2} \int_{J_0}^{J} \frac{\mathrm{d}J_1}{J_1} \int_{J_0}^{J_1} \mathrm{d}J_2 \frac{(1+J_2)^{2\mu\sigma^{-2}+2}}{(1+J_1)^{2\mu\sigma^{-2}+3}} .$$

Integrating with respect to J_2 and assuming $J_0 \to 0$, one obtains

$$T(0, J) = \frac{\sigma^{-2}}{\mu\sigma^{-2} + 3/2} \int_0^J \frac{\mathrm{d}z}{z} \left(1 - \frac{1}{(1+z)^{2\mu\sigma^{-2}+3}}\right) . \tag{10}$$

The latter equation is identical to Eq. (13) in the main paper. For the integral in Eq. (10), one can consider the convergence properties near $J = 0$ and $J \to +\infty$ and explicitly write

$$T(0, J) = \frac{1}{\sigma^2} \left[\frac{\ln(1+J)}{\mu\sigma^{-2} + 3/2} + \frac{J^{-(2\mu\sigma^{-2}+3)}}{2(\mu\sigma^{-2} + 3/2)^2} + \tau\left(\frac{2\mu}{\sigma^2}, J\right) \right], \tag{11}$$

where $\tau(q, J)$ is small compared to the sum of the first and second terms in the brackets for $J \gg 1$. For $2\mu\sigma^{-2} + 3 > 0$, the first passage time is logarithmically large $\propto \ln J$, meaning that the synchronous state attracts the system trajectories averagely or from time to time (during intermittent epoches of synchronization). For $2\mu\sigma^{-2} + 3 < 0$, the first passage time diverges as a power law of J, meaning that the synchronous state is strongly repelling and the passages of the system trajectories near it are rare events.

III. AVERAGE FREQUENCY

In this section we perform a detailed calculation of the average frequency of oscillations. Technically, our task is to derive the mean frequency for Eq. (17) of the main paper. Eq. (17) of main paper

$$\dot{\theta} = \omega - \mu b \sin\theta - \frac{\sigma^2}{2} c \sin\theta - \frac{\sigma}{\sqrt{2}} \sin\theta \, \zeta_1(t) + \frac{\sigma}{\sqrt{2}} (\cos\theta - c) \, \zeta_2(t) \tag{12}$$

yields the Fokker–Planck equation

$$\frac{\partial w(\theta,t)}{\partial t} + \frac{\partial}{\partial\theta} \left[\left(\omega - \mu b \sin\theta - \frac{\sigma^2}{2} c \sin\theta \right) w(\theta,t) \right] - \frac{\sigma^2}{2} \frac{\partial}{\partial\theta} \left(\sin\theta \frac{\partial}{\partial\theta} \left(\sin\theta \, w(\theta,t) \right) \right)$$
$$- \frac{\sigma^2}{2} \frac{\partial}{\partial\theta} \left((\cos\theta - c) \frac{\partial}{\partial\theta} \left((\cos\theta - c) \, w(\theta,t) \right) \right) = 0 ,$$

which can be simplified to

$$\frac{\partial w(\theta,t)}{\partial t} + \frac{\partial}{\partial\theta} \left[\left(\omega - \mu b \sin\theta \right) w(\theta,t) \right] - \frac{\sigma^2}{2} \frac{\partial^2}{\partial\theta^2} \left[\left(1 + c^2 - 2c\cos\theta \right) w(\theta,t) \right] = 0 . \tag{13}$$

For a time-independent distribution $w(\theta)$, one can integrate the latter equation with respect to θ and obtain

$$(a - \mu b \sin\theta) w - \frac{\sigma^2}{2} \frac{\mathrm{d}}{\mathrm{d}\theta} \left[(1 + c^2 - 2c\cos\theta) w \right] = j , \tag{14}$$

where $j = \mathit{const}$ is the integration constant, which is the probability flux in the system. Flux j counts the average number of crossings of certain state θ_0 per the unit time or, which is the same, number of phase turnovers per the unit time. Hence, $\langle\dot{\theta}\rangle = 2\pi j$.

A. Continuous fraction expansion for Eq. (18) of the main article

Let us use the Fourier expansion for $w(\theta)$,

$$w(\theta) = \frac{1}{2\pi} \sum_{k=-\infty}^{+\infty} w_k e^{ik\theta} \,,$$

and substitute into Eq. (14);

$$\Big(a - \mu b \frac{e^{i\theta} - e^{-i\theta}}{2i}\Big) \sum_k w_k e^{ik\theta} - \sigma^2 \sum_k ik w_k \frac{1+c^2}{2} e^{ik\theta} + \sigma^2 c \frac{d}{d\theta} \sum_k \frac{e^{i\theta} + e^{-i\theta}}{2} w_k e^{ik\theta} = 2\pi j \,.$$

After recollecting terms, one finds

$$\sum_k e^{ik\theta} \left[a w_k - \frac{\mu b}{2i} w_{k-1} + \frac{\mu b}{2i} w_{k+1} - ik\sigma^2 \frac{1+c^2}{2} w_k + \frac{ik\sigma^2 c}{2} (w_{k-1} + w_{k+1}) \right] = j \,.$$

It is more convenient to consider separately $k = 0$ and $k > 0$ and bear in mind that $w_0 = 1$, $w_{-k} = w_k^*$. For $k = 0$,

$$a - \frac{\mu b}{2i} w_1^* + \frac{\mu b}{2i} w_1 = 2\pi j$$

or

$$2\pi j = \langle \dot{\theta} \rangle = a + \mu b \, \mathrm{Im}(w_1) \,, \tag{15}$$

for $k \geq 1$,

$$w_{k-1} \left[-\frac{\mu b}{2i} + \frac{ik\sigma^2 c}{2} \right] + w_k \left[a - ik\sigma^2 \frac{1+c^2}{2} \right] + w_{k+1} \left[\frac{\mu b}{2i} + \frac{ik\sigma^2 c}{2} \right] = 0 \,. \tag{16}$$

Introducing the ratio $w_{k-1}/w_k = r_k$, one can express from Eq. (16)

$$r_k = \frac{1 + c^2 + 2ia/k}{c + \mu b/k} - \frac{c - \mu b/k}{c + \mu b/k} \frac{1}{r_{k+1}} \,. \tag{17}$$

By definition of r_k,

$$w_1 = \frac{1}{r_1} \,.$$

Eq. (17) is a recurrence relation which allows one to calculate $w_1 = 1/r_1$ as a continuous fraction;

$$w_1 = \cfrac{1}{A_1 - B_1 \cfrac{1}{A_2 - B_2 \cfrac{1}{A_3 - B_3 \cfrac{1}{\dots}}}} \,, \tag{18}$$

where $A_k \equiv \dfrac{1 + c^2 + 2ia/k}{c + \mu b/k}$ and $B_k \equiv \dfrac{c - \mu b/k}{c + \mu b/k}$. For to achieve a faster convergence of truncated continuous fractions, one can notice that $A_\infty = 1/c + c$, $B_\infty = 1$ and, according to Eq. (17), $r_\infty = c$ (the second solution $r_\infty = 1/c$ corresponds to a diverging expansion for $c < 1$). The truncation error is minimal for r_∞ substituted in place of truncated r_k in Eq. (18). With calculated w_1, one evaluate the average frequency from Eq. (15). The results of calculation of $\langle \dot{\theta} \rangle$ are plotted in Fig. 2 of the main article.

IV. DIRECT NUMERICAL SIMULATION FOR ENSEMBLES OF PHASE OSCILLATORS

In Fig. 3 of the main paper, the results of direct numerical simulation for the ensemble of the phase oscillators [Eq. (1) of the main paper] are plotted. For a finite number N of oscillators in the ensemble, the order parameter $R\, e^{i\Phi} = N^{-1} \sum_{j=1}^{N} e^{i\varphi_j}$. For the stochastic phase equations written in the Stratonovich form we employed 4-stage stochastic Runge–Kutta method suitable for the case of non-commutative noise; the method is described in [P.M. Burrage, *Runge–Kutta methods for stochastic differential equations*, PhD Thesis (University of Queensland, Brisbane, Australia, 1999)]. The strong order of the method is 1.5 for non-commutative noise, for vanishing noise, the method turns into a classical deterministic 4-stage Runge–Kutta method of order 4.
